# A hierarchical federated learning framework with FedNova, game-theoretic matching, and QKD-assisted privacy for the internet of vehicles

**DOI:** 10.3389/frai.2026.1861374

**Published:** 2026-07-29

**Authors:** L. Jai Vinita, V. Vetriselvi

**Affiliations:** 1 School of Computer Science and Engineering, Vellore Institute of Technology, Chennai, Tamil Nadu, India; 2 Department of Computer Science and Engineering, College of Engineering, Anna University, Chennai, India

**Keywords:** client selection, federated learning, game-theoretic, heterogeneous data, Internet of Vehicles, privacy

## Abstract

The Internet of Vehicles (IoV) supports essential intelligent transportation applications but encounters challenges in federated learning (FL) due to non-independent and identically distributed (non-IID) data, vehicle mobility, resource heterogeneity, and strict privacy requirements in latency-sensitive scenarios such as misbehavior detection and accident response. Traditional FL methods, such as random client selection and standard FedAvg, often experience slow convergence and reduced performance under non-IID conditions. We introduce a hierarchical federated learning framework for software-defined vehicular fog computing. The framework incorporates FedNova (a normalized-averaging aggregation method for heterogeneous federated optimization) to produce normalized model updates under data heterogeneity, a Reward-Based Payoff Strategy (RBPS) for incentive-aware client selection, and game-theoretic vehicle-aggregator matching based on the college admissions problem. Privacy is strengthened through quantum key distribution (QKD)-assisted secure key establishment and classical gradient masking, with quantum circuit simulation used to assess future enhancements. The three-layer architecture includes vehicles, Roadside Unit (RSU)/ Base Station (BS)-level aggregators, and a Software-Defined Network Controller (SDNC) global aggregator. The framework uses both monetary and service-based incentives, such as toll exemptions, to encourage vehicle participation. Hybrid simulations using OMNeT++, Veins, SUMO, and the VeReMi misbehavior detection dataset show that the proposed approach achieves 94.8% classification accuracy [95% Confidence Interval (CI): 92.7–97.0 over 10 runs], converges in 120 rounds (33% faster than FedAvg), and reduces average latency by 29% (320 ms compared to 450 ms for FedAvg), with statistically significant improvements (*p* < 0.05). These gains enable faster model adaptation to evolving attacks (5–10 min shorter training cycles) and support real-time safety applications where delays above 400 ms can compromise road safety. Ablation studies confirm the complementary roles of FedNova, RBPS, and matching. Although quantum operations are currently simulated classically, the design remains compatible with future quantum hardware.

## 1 Introduction

The Internet of Vehicles (IoV) serves as a foundational element of next-generation intelligent transportation systems, facilitating vehicle-to-everything (V2X) communication, real-time traffic management, autonomous driving support, and misbehavior detection (; Tomkos et al., 2020; Alwis et al., 2021). The transition to 5G and 6G networks has introduced stringent requirements for ultra-low latency and high reliability (Alwis et al., 2021). Despite these advancements, IoV applications face significant challenges, including high vehicle mobility, heterogeneous onboard computing resources, non-independent and identically distributed (non-IID) data, and strict data privacy regulations.

Centralized machine learning approaches have become increasingly impractical in IoV contexts due to communication overhead, vulnerability to single points of failure, and heightened privacy risks such as model inversion and membership inference attacks (McMahan et al., 2017; Rasheed et al., 2022; Wazzeh et al., 2023). Federated Learning (FL) addresses these concerns by enabling collaborative model training while retaining raw vehicle data (McMahan et al., 2017). However, conventional FL algorithms, including FedAvg, encounter substantial obstacles in IoV environments, such as slow convergence with non-IID data, inefficient random client selection, elevated communication costs, and limited incentives for participation by resource-constrained vehicles (Abdulrahman et al., 2021b; Tu et al., 2022).

Recent research has investigated customized client selection strategies (Nishio and Yonetani, 2019; Kang et al., 2020a), heterogeneity-aware aggregation methods such as FedNova (Wang et al., 2020), game-theoretic task allocation (Chen et al., 2021), and privacy-enhancing techniques, including differential privacy and secure aggregation (Moya Osorio et al., 2022; Sumitra and Shenoy, 2023). Additionally, emerging studies are exploring quantum-assisted federated learning, leveraging quantum key distribution (QKD) for secure key exchange and quantum-inspired approaches to strengthen privacy protection (Qu et al., 2024; Innan et al., 2024; Hazarika et al., 2025).

### 1.1 Challenges in federated learning for latency-critical IoV applications

Federated learning in the Internet of Vehicles faces four intertwined challenges that render conventional approaches inadequate for safety-critical applications such as misbehavior detection and real-time accident response. First, **data heterogeneity** arises because vehicles encounter highly non-IID distributions—local datasets differ significantly in traffic density, road conditions, and attack patterns—leading to objective inconsistency and slow convergence under standard FedAvg aggregation (Wang et al., 2020). Second, **high vehicle mobility** causes frequent topology changes, unstable V2X links (especially under varying Received Signal Strength Indicator (RSSI) levels), and potential client dropouts, complicating reliable client selection and aggregation. Third, **resource heterogeneity** (onboard CPU, memory, residual energy, and communication interfaces such as LTE vs. IEEE 802.11p) results in stragglers and unfair participation if incentives are ignored. Fourth, **strict privacy and latency requirements** demand protection against gradient inversion attacks (e.g., Deep Leakage from Gradients) while keeping end-to-end latency below 300–400 ms to support time-sensitive decisions.

These challenges motivate our design choices: **FedNova** normalizes updates to mitigate non-IID effects (Wang et al., 2020); the **Reward-Based Payoff Strategy (RBPS)** jointly evaluates local model quality, residual energy, and privacy cost for incentive-aware client selection; **game-theoretic matching** inspired by the college admissions problem ensures stable, mobility-aware vehicle-aggregator pairings (Gale and Shapley, 1962); and **QKD-assisted key establishment** combined with classical gradient masking strengthens privacy without excessive computational overhead. The resulting hierarchical three-layer software-defined vehicular fog architecture [vehicles → Roadside Unit (RSU)/Base Station (BS) Level-1/Level-2 aggregators → Software-Defined Network Controller (SDNC) global aggregator] distributes computation, reduces central bottlenecks, and aligns incentives through monetary and service-based rewards (e.g., toll exemptions). This integrated approach addresses the limitations of prior FL methods that typically tackle only one or two of these issues in isolation.

This study introduces a comprehensive hierarchical federated learning framework tailored for software-defined vehicular fog computing, progressing from the four challenges identified above to a concrete architecture (Section 3), an incentive-aware selection and matching mechanism, a hybrid quantum-classical privacy layer, and an extensive experimental evaluation (Section 4) that validates each design choice individually and in combination.

The primary contributions of this work are as follows:

- We introduce the first (to our knowledge) tightly coupled integration of FedNova normalized aggregation, a multi-criteria Reward-Based Payoff Strategy (RBPS), and game-theoretic stable matching (based on the college admissions problem) within a hierarchical three-layer software-defined vehicular fog architecture. Unlike previous studies that address these techniques in isolation, our framework creates a closed feedback loop in which RBPS payoffs—jointly considering local model quality, residual energy, and privacy cost—directly drive mobility-aware vehicle-aggregator pairings, while FedNova ensures robust convergence under severe non-IID data distributions.
- A practical hybrid quantum-classical privacy mechanism that combines QKD-derived keys for secure key establishment with classical gradient masking, specifically designed to meet the stringent latency and privacy requirements of safety-critical IoV applications such as misbehavior detection.
- A scalable three-layer fog architecture (vehicles → RSU/BS Level-1/Level-2 aggregators → SDNC) that performs preliminary RBPS computations and matching at the edge to reduce latency and central bottlenecks while supporting dynamic vehicle mobility.
- Extensive performance evaluation on the VeReMi misbehavior detection dataset using realistic OMNeT++ + Veins + SUMO simulations, demonstrating superior results (94.8% accuracy, 33% faster convergence, 29% lower latency, and significantly reduced privacy leakage) compared to strong baselines. Ablation studies and RSSI-perturbed sensitivity analysis confirm the complementary benefits of the proposed integration.

The remainder of the paper is organized as follows. Section 2 reviews related work in federated learning for IoV, client selection, matching theory, and privacy mechanisms. Section 3 describes the proposed framework in detail. Performance evaluation is presented in Section 4, followed by discussion and conclusions in Sections 4.6 and 5.

## 2 Related work

Federated learning within the Internet of Vehicles must address challenges, including vehicle mobility, stringent latency requirements, non-independent and identically distributed (non-IID) data, and privacy risks in applications such as misbehavior detection and real-time accident response. This section reviews significant advances in FL for IoV, including client selection, task allocation, fog and edge architectures, and privacy and security mechanisms. The discussion positions the proposed hierarchical framework, which integrates FedNova, incentive-aware selection through the Reward-Based Payoff Strategy (RBPS), game-theoretic matching, and quantum key distribution (QKD)-assisted secure aggregation.

### 2.1 Federated learning in IoV

FL has become essential for privacy-preserving IoV applications, including the detection of misbehavior and Sybil attacks (Vinita and Vetriselvi, 2023). Comprehensive surveys outline FL's advantages over centralized learning while highlighting challenges in heterogeneity, communication overhead, and dynamic topologies (Abdulrahman et al., 2021b; Kairouz et al., 2021). Recent IoV-specific works include AI-enhanced FL for cyberthreat detection with differential privacy (Alghamdi et al., 2025), flexible aggregation reducing energy consumption (Qayyum et al., 2023), and explainable and secured quantum FL (ESQFL) for smart-grid-related security in vehicular settings (Ren et al., 2025). Emerging quantum federated learning (QFL) approaches integrate quantum techniques to enhance privacy and efficiency. Qu et al. (2024) proposed QFSM, a quantum minimal gated unit-based method for speech emotion recognition in 5G IoV, reporting improved accuracy and privacy. Kim et al. (2023) explored QFL for vehicular computing scenarios, focusing on distributed quantum advantages. More recent efforts include quantum-enhanced FL for metaverse-empowered vehicular networks (Hazarika et al., 2025) and adaptive-trust QFL frameworks for secure IoV (Alruwaili and Islam, 2024). Comprehensive QFL surveys (Sai et al., 2025; Nguyen et al., 2025) discuss hybrid quantum-classical architectures, Noisy Intermediate-Scale Quantum (NISQ) limitations, and applications in vehicular and edge networks. Unlike these works, which often focus on either classical optimization or standalone quantum components, our framework integrates FedNova normalization, incentive-driven selection, and hierarchical fog aggregation tailored to latency-critical misbehavior detection.

### 2.2 Client selection methods

Efficient client selection remains essential in resource-constrained and mobile Internet of Vehicles (IoV) environments. Early approaches, including probability-based methods (Goetz et al., 2019) and the Power-of-Choice algorithm (Cho et al., 2020), enhance convergence rates but rely on the assumption of static client availability. FedCS (Nishio and Yonetani, 2019) incorporates resource considerations to reduce latency, while multi-criteria frameworks such as FedMCCS (Abdulrahman et al., 2021a) and fairness-aware schemes (Huang et al., 2020, 2022) address system heterogeneity.

Incentive-aware and heterogeneity-focused strategies encompass RBPS-style payoff mechanisms that balance accuracy, energy consumption, and privacy costs (Kang et al., 2020a), as well as dynamic scoring (Yang et al., 2022), group customization (Hu et al., 2023), and clustered client selection (Sattler et al., 2021). Recent IoV-specific developments include fairness-aware selection (Mughal et al., 2024) and genetic algorithm-optimized multi-objective strategies that reduce communication delay (Liu et al., 2024).

The proposed RBPS approach extends existing methods by incorporating mutual preferences through game-theoretic matching, which ensures stable and fair vehicle-aggregator pairings in dynamic environments.

### 2.3 Task allocation and fog computing

Matching theory has been extensively utilized for task allocation and resource scheduling in the Internet of Vehicles (IoV). Reputation-based approaches (Kang et al., 2020b), bilateral mechanisms (Wehbi et al., 2023), and multi-channel game-theoretic selection (Ding et al., 2025) have demonstrated reductions in latency. Additionally, energy-aware strategies (Albelaihi et al., 2022; Tan et al., 2025) enhance power optimisation in edge environments. Vehicular fog computing further enables low-latency aggregation through offloading (Xiao and Zhu, 2017; Arisdakessian et al., 2020) and cooperative frameworks (Sodhro et al., 2020; Luo et al., 2020).

The proposed three-layer software-defined fog architecture extends these concepts by employing hierarchical aggregation and college-admissions-inspired matching to balance load, minimize latency, and align incentives.

### 2.4 Security considerations

Security is a critical concern in the Internet of Vehicles (IoV) (Luong et al., 2020; Wu et al., 2020). Traditional methods such as differential privacy (Moya Osorio et al., 2022) and blockchain-enhanced federated learning (FL) (Kim et al., 2020) have been employed, while secure aggregation addresses inference risks (Sumitra and Shenoy, 2023). However, regularization-based techniques like FedProx often result in reduced accuracy when applied to non-independent and identically distributed (non-IID) data (Abdulrahman et al., 2021b). Quantum-based methods demonstrate significant potential for future security enhancements. For example, FedQNN (Innan et al., 2024) and hybrid quantum key distribution (QKD) frameworks (Sawaika et al., 2025) have reported reductions in information leakage within distributed environments. In IoV-specific applications, QFSM (Qu et al., 2024) and metaverse-oriented quantum federated learning (QFL) (Hazarika et al., 2025) utilize quantum circuits and QKD to strengthen privacy protections.

This work adopts quantum key distribution (QKD) for secure key exchange, combined with classical gradient masking and simulated quantum circuits for exploratory purposes. This approach offers practical privacy enhancements and maintains compatibility with existing hardware. Unlike purely quantum-based proposals, the focus here is on solutions that are deployable within current IoV fog infrastructures.

Table 1 demonstrates that although recent QFL studies have advanced privacy and task-specific performance, few approaches incorporate heterogeneity management (FedNova), incentive mechanisms, and stable matching within a fog-based IoV architecture for latency-sensitive applications. This integration represents the primary distinction of the present work.

**Table 1:** Comparison of recent QFL approaches in vehicular/IoV contexts.

Work	Year	Architecture	Quantum component	IoV focus	Key advantage/limitation
Kim et al. (2023)	2023	Hybrid	Quantum model training	Vehicular computing	Early exploration; limited evaluation
Qu et al. (2024)	2024	Federated	Quantum minimal gated unit	Speech emotion in 5G IoV	Accuracy + privacy; task-specific
Hazarika et al. (2025)	2025	Decentralized	QNN + QKD	Metaverse vehicular	Heterogeneity-aware; metaverse-oriented
Alruwaili and Islam (2024)	2024	Adaptive trust	Quantum components	Secure IoV networks	Trust modeling; conference paper
Our work	–	Hierarchical fog	QKD + classical masking (sim. quantum)	Misbehavior detection	FedNova + RBPS + matching; latency-focused

Beyond the quantum-assisted line of work, three very recent contributions target classical federated intrusion detection for IoV directly, all from the same research group. Heidari et al. (2026c) propose FedIoV, which combines encrypted client-server communication with a two-stage robust aggregation scheme (TOPSIS-based dimensionality reduction and Multi-Krum clustering) to filter noisy or malicious updates, a lightweight interpretable classifier (KANConvNet) inspired by the Kolmogorov-Arnold theorem, and genetic-algorithm-driven hyperparameter tuning across clients. The same group's FLITE (Heidari et al., 2026b) instead uses a deep-reinforcement-learning scheduler at RSUs to select clients and transmit power based on data quality, channel state, and device energy, while their VITA framework (Heidari et al., 2026a) targets self-supervised, label-free intrusion detection using an adversarial autoencoder with channel attention and residual temporal convolutional networks. Together, these three works reflect the field's continued emphasis on robust aggregation, energy-aware scheduling, and label-free detection for vehicular intrusion detection, complementing the incentive- and matching-based client selection mechanism proposed here.

### 2.5 Positioning of our work

While recent studies have advanced individual aspects of FL for IoV—such as heterogeneity-aware aggregation (FedNova), incentive mechanisms, game-theoretic task allocation, and quantum-enhanced privacy—the proposed framework distinguishes itself through the *tight synergistic integration* of these elements within a practical hierarchical fog architecture tailored to latency-critical misbehavior detection. It is important to distinguish this integration from prior work on hierarchical federated learning for vehicular networks in isolation. For instance, HaghighiFard and Coleri (HaghighiFard and Coleri, 2024) propose a hierarchical FL framework over multi-hop clustered VANETs that groups vehicles by a weighted combination of relative speed and model-parameter cosine similarity to handle mobility and non-IID data; this clustering criterion optimizes for cluster-head stability alone and includes neither an incentive mechanism nor a formal matching game, so vehicle-aggregator pairings are not jointly optimized against energy, accuracy, and privacy objectives the way RBPS-driven matching is here. Similarly, other hierarchical FEEL-style architectures typically fix the vehicle-to-aggregator assignment structurally (e.g., by geographic cell or fixed edge-cloud tiers) rather than deriving it from a game-theoretic stable-matching process, and none of the reviewed hierarchical frameworks combine heterogeneity-aware normalization (FedNova), incentive-aware selection, and QKD-assisted privacy in a single pipeline.

Specifically:

- Unlike hierarchical FL frameworks that fix vehicle-aggregator assignment by clustering or fixed network tiers alone (e.g., HaghighiFard and Coleri, 2024), our architecture derives assignment from a two-sided stable matching game whose preferences are driven by the RBPS payoff, jointly accounting for accuracy, energy, and privacy rather than mobility or similarity metrics alone.
- Unlike standalone FedNova or FedProx deployments, we couple normalized aggregation with a multi-criteria **Reward-based payoff strategy (RBPS)** that explicitly balances local accuracy, residual energy, and privacy overhead, feeding directly into a two-sided stable matching game. This creates a closed feedback loop between incentives, selection, and pairing that accounts for vehicle mobility and aggregator capacity.
- Privacy protection goes beyond classical differential privacy or secure aggregation by employing **QKD-derived keys** for encryption and classical gradient masking, with hierarchical aggregation to minimize central exposure. While related QFL works focus on task-specific quantum models or pure quantum neural networks, our design remains deployable on current hardware (quantum circuits are simulated classically for exploratory phase obfuscation) while ensuring compatibility with near-term quantum devices.
- The three-layer software-defined architecture performs preliminary RBPS computations and matching at Level-1 aggregators (RSUs/BSs), enabling low-latency adaptation to mobility and channel conditions—extending beyond flat or purely edge-based designs.

To the best of our knowledge, this is the first work to jointly optimize FedNova normalization, incentive-driven RBPS selection, college-admissions-inspired stable matching, and hybrid quantum-classical privacy in a unified fog-based FL pipeline for IoV misbehavior detection on the VeReMi dataset. This integration yields complementary gains in accuracy, convergence speed, latency, and privacy that exceed the sum of individual components, as validated by ablation studies.

### 2.6 Recent advances in multi-objective optimization and reliability engineering

Recent studies have significantly advanced multi-objective optimization and reliability engineering in industrial systems. Djami and Nzié (2025) proposed a meta-modeling approach using evolutionary algorithms for predictive maintenance of a corn sheller, achieving high prediction accuracy (R^2^ = 0.92) and substantial cost savings. Similarly, Djami et al. (2024a) applied the NSGA-II algorithm for multi-objective optimization of cutting parameters in hard turning, demonstrating effective Pareto front capture.

In reliability and risk analysis, Djami et al. (2026a,b) developed hybrid evolutionary and stochastic methods to optimize the dependability of a hammer mill under budgetary constraints, while Djami et al. (2024b) utilized particle swarm optimization for diagnosis and prognosis of soybean roaster failures. Complementary work by the same research group addressed residual reliability of reinforced concrete under buckling loads (Djami et al., 2024c) and system reliability evaluation using Monte Carlo simulation (Djami et al., 2024d).

Our proposed framework builds upon these advances by integrating evolutionary optimisation principles into a hierarchical federated learning architecture. Unlike the above works that primarily target single-machine or centralized industrial systems, our approach addresses distributed, non-IID data, mobility, and privacy challenges in dynamic IoV environments through FedNova normalization, incentive-aware RBPS selection, and game-theoretic matching. This synergistic integration offers a novel contribution tailored for latency-critical vehicular intelligence.

## 3 Proposed framework

**Figure 1:**
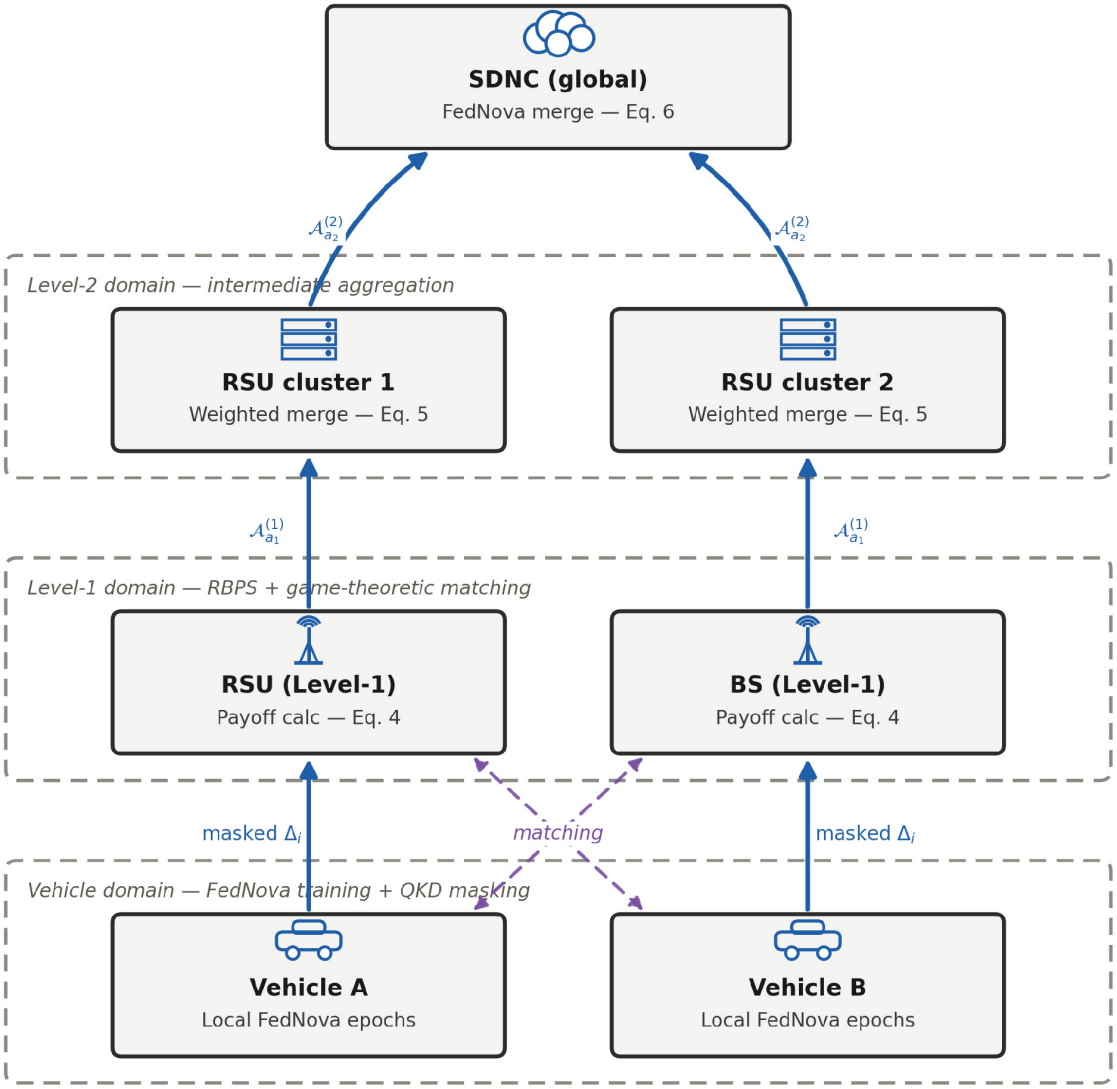
Proposed three-layer vehicular fog architecture for privacy-preserving FL in IoV. Solid blue arrows show the upward masked-model-update path with the corresponding equation from Section 3.2; dashed purple arrows show that vehicle-aggregator pairing is determined by the game-theoretic matching of Section 3.4 rather than fixed by column position. Dashed gray boxes group blocks by functional domain.

This section presents a scalable federated learning framework for the Internet of Vehicles (IoV) that integrates FedNova to address non-independent and identically distributed (non-IID) data, a Reward-Based Payoff Strategy (RBPS) for incentive-aware client selection, and game-theoretic matching inspired by the college admissions problem. The framework operates within a three-layer software-defined vehicular fog architecture, under the assumption of reliable LTE/802.11p communication and no client dropouts per round. Future work will incorporate models for disconnections and handovers to improve mobility management (Figure 1).

Future extensions will incorporate realistic models for client dropouts, link disconnections, and handovers caused by high vehicle mobility. In the current work, we adopt the simplifying assumption of no dropouts per round (common in many initial FL simulation studies) to focus on the core integration of FedNova normalization, RBPS incentive mechanisms, game-theoretic matching, and QKD-assisted privacy. This allows us to isolate and evaluate the synergistic benefits of the proposed components under controlled conditions. Robustness to channel quality variations (RSSI) is already demonstrated through the expanded sensitivity analysis (see Section 4).

It is important to note that quantum key distribution and exploratory quantum circuit simulations (implemented via Qiskit) are performed classically in the current implementation. The framework is designed as a hybrid quantum-classical system that provides immediate practical benefits through classical gradient masking while remaining fully compatible with near-term quantum hardware for enhanced key establishment and phase-based obfuscation.

The architecture is structured as follows:

- **Bottom layer (vehicles)**: Vehicles perform local training using FedNova, producing normalized model updates to mitigate non-IID heterogeneity. Updates are encrypted with keys from quantum key distribution (QKD) and masked classically to obfuscate gradients.
- **Middle layer (level-1 and level-2 aggregators)**: Level-1 aggregators (RSUs/BSs) compute preliminary RBPS payoffs and apply adaptive game-theoretic matching for efficient vehicle-aggregator pairings, improving latency and resource use. Level-2 aggregators (RSU Clusters) conduct intermediate aggregation of masked updates.
- **Top layer (SDNC)**: The Software-Defined Network Controller functions as the global aggregator, conducting the final FedNova-weighted aggregation. Privacy is preserved using QKD-derived keys for decryption, followed by classical masking removal and re-masking of the global model prior to distribution. Quantum circuit simulations, such as those implemented with Qiskit, are used to investigate potential phase-based obfuscation for future enhancements.

### 3.1 Communication protocol, scheduling, and synchronization

The framework operates as a *synchronous* federated learning system: all three aggregation stages (vehicle-to-Level-1, Level-1-to-Level-2, Level-2-to-SDNC) complete within a single communication round before the next round begins, and the global model update frequency is exactly one update per round. Under the no-dropout assumption stated above, every selected vehicle's update is included in its round's aggregation; asynchronous or partial-participation variants are left to future work (Section 4.6).

Each communication round proceeds as follows:

1. **Broadcast (top-down):** The SDNC broadcasts the current global model to all Level-2 (RSU Cluster) aggregators over the wired SDN control-plane backhaul, which in turn forward it to their subordinate Level-1 (RSU/BS) aggregators; each Level-1 aggregator broadcasts the model to its currently matched vehicles over LTE or IEEE 802.11p, consistent with the communication interfaces in Table 3.
2. **Local training:** Each vehicle performs *n*_*i*_ local FedNova training epochs (5 local epochs per round at batch size 32 in our experiments) on its local, non-IID data partition.
3. **Secure upload:** Vehicles encrypt their normalized local updates using QKD-derived keys (256-bit key size, Table 3) and apply classical gradient masking before transmitting to their matched Level-1 aggregator over the vehicle's wireless interface.
4. **Level-1 aggregation and matching:** Each Level-1 aggregator computes preliminary RBPS payoffs for its associated vehicles, performs (or updates) the game-theoretic matching described in Section 3.2, and aggregates the masked updates it received in this round using the accuracy- and data-volume-weighted scheme of Equation 4.
5. **Level-2 aggregation:** Level-1 aggregators forward their partially aggregated, still-masked models to their assigned Level-2 (RSU Cluster) aggregator over the wired backhaul; Level-2 performs intermediate aggregation (Equation 5) once per round, after all of its Level-1 aggregators have reported.
6. **Global aggregation:** The SDNC collects all Level-2 outputs, removes classical masking using QKD-derived keys, performs the final FedNova-weighted global aggregation (Equation 6), re-masks the resulting global model, and the round repeats from step 1.

**Table 2:** Detailed parameter settings for reproducibility.

Parameter	Value/description
Number of vehicles (*C*)	50
Number of selected FL vehicles per round	50 (all vehicles participate)
Number of level-1 Aggregators	5 (RSUs/BSs)
Number of level-2 Aggregators	2 (RSU clusters)
Non-IID data distribution	Dirichlet distribution (α = 0.1)
Neural network architecture	3 convolutional layers + 2 fully connected layers
Optimizer	Adam
Initial learning rate	0.001
Local batch size	32
Local epochs per round	5
Total communication rounds	200
RSSI range (LTE & 802.11p)	[–130, –20] dBm
Residual energy range	[0, 90] J
Memory capacity range	[4, 7068] KB
Data records per vehicle	[0, 4000] bits
Model parameter size	5 MB
QKD key size (simulated)	256 bits
Simulation platforms	OMNeT++ 5.6.2 + Veins 5.2 + SUMO 1.9.2
FL framework	PyTorch 1.8.1 + Flower
Quantum simulator	Qiskit 1.0.2 (classical simulation)
Random seed	42 (for reproducibility)

**Table 3:** Notation table.

Symbol	Description
*acc*_*i*_	Local accuracy
*B*_*i*_	Residual energy (joules)
*P*_*i*_	Privacy-preservation contribution (masking effort)
*w*_*a*_, *w*_*b*_, *w*_*p*_	Weights for accuracy, energy, privacy
*U*_*i*_	RBPS payoff function
θ_*i*_	Threshold for client selection (0.7)
Δ_*i*_	Local model update
*n*_*i*_	Number of local iterations
V	Set of vehicles
A	Set of aggregators
*R*_*v*_	Vehicle reward
σ_*va*_	Pairing cost
$I_{v}^{\text{comp}}$	Computation incentive
κ_*v*_	Scaling factor
μ_*v*_	CPU cycles
α	Computation coefficient
δ_*v*_	Data size
β	Data coefficient
ε_*v*_	Model parameters
γ	Parameter coefficient
$I_{v}^{\text{comm}}$	Communication incentive
τ_*va*_	Transmission cost
$\lambda_{v}^{LTE}$	LTE bandwidth
θ	LTE coefficient
$\lambda_{v}^{802.11p}$	802.11p bandwidth
η	802.11p coefficient

#### 3.1.1 Communication protocol assumptions

Vehicle-to-Level-1 links use LTE or IEEE 802.11p, both explicitly modeled with RSSI-dependent bandwidth (RSSI range in Table 3; bandwidth notation $\lambda_{v}^{LTE}$, $\lambda_{v}^{802.11p}$ in Table 2); Level-1-to-Level-2 and Level-2-to-SDNC links are assumed to run over a reliable, low-latency wired SDN control-plane backhaul (consistent with the software-defined vehicular fog design), so packet loss and queuing delay are considered negligible above the RSU layer and are not separately modeled in this work.

#### 3.1.2 Matching and RBPS update frequency

Preliminary RBPS payoffs and vehicle-aggregator matching are recomputed once per communication round by default, keeping pairings aligned with each round's channel conditions and vehicle positions. In addition, an out-of-cycle re-matching is triggered whenever RSSI degrades beyond a threshold or a vehicle's connectivity to its current Level-1 aggregator is lost, as discussed in Sections 4.5, 4.6; this event-triggered re-matching operates independently of the fixed per-round schedule to react faster to sudden mobility or channel changes than the round boundary alone would allow.

### 3.2 Reward-based payoff strategy (RBPS) and vehicle-aggregator matching

The Reward-Based Payoff Strategy (RBPS) is defined as *U*_*i*_ = *w*_*a*_·*acc*_*i*_ + *w*_*b*_·*B*_*i*_ − *w*_*p*_·*P*_*i*_, where *P*_*i*_ represents the privacy cost (masking overhead). The negative sign ensures that higher privacy cost reduces the payoff, encouraging vehicles to balance utility with privacy protection. Weights *w*_*a*_ = 0.5, *w*_*b*_ = 0.3, *w*_*p*_ = 0.2 were determined through grid search. This formulation provides a clear multi-objective trade-off and directly feeds into the game-theoretic matching process.

The RBPS payoff function, *U*_*i*_ = *w*_*a*_·*acc*_*i*_ + *w*_*b*_·*B*_*i*_ + *w*_*p*_·*P*_*i*_, integrates local accuracy (*acc*_*i*_), residual energy (*B*_*i*_), and privacy cost (*P*_*i*_), with the weights *w*_*a*_, *w*_*b*_, and *w*_*p*_ optimized through grid search (initial values: *w*_*a*_ = 0.5, *w*_*b*_ = 0.3, *w*_*p*_ = 0.2). Preliminary calculations of *U*_*i*_ are performed at Level-1 aggregators to alleviate bottlenecks and accommodate mobility, while final selections are made at the SDNC. Privacy is strengthened through QKD-assisted key establishment for secure aggregation combined with classical gradient masking. It should be noted that all quantum operations, including key distribution and exploratory-phase obfuscation circuits, are currently performed via classical simulation with Qiskit. The framework is explicitly designed as a hybrid quantum-classical system that delivers immediate practical privacy benefits while remaining compatible with future quantum hardware.

Privacy protection is evaluated under a semi-honest adversary model, where aggregators follow the protocol but may attempt to infer private information from received updates. The main attack considered is Deep Leakage from Gradients (DLG), with success rate reduced to 5.0% compared to 25.0% for FedAvg. Future work will extend the evaluation to additional attacks, including membership inference, property inference, and malicious aggregator collusion scenarios.

### 3.3 Vehicle optimization

Each vehicle acts as a rational agent aiming to maximize its individual reward while considering the cost of pairing with an aggregator. The reward function is defined as (Equation 1–Equation 3):


$$\begin{array}{l} R_{v}=\left(1-\sigma_{va}\right)\cdot \left(I_{v}^{\text{comp}}+I_{v}^{\text{comm}}\right) \end{array}$$ (1)

where σ_*va*_ ∈ [0, 1] is the normalized pairing cost (accounting for mobility, distance, and signal quality), $I_{v}^{\text{comp}}$ is the computation incentive, and $I_{v}^{\text{comm}}$ is the communication incentive.

The computation incentive is given by:


$$\begin{array}{l} I_{v}^{\text{comp}}=\kappa_{v}\cdot \left(\mu_{v}\cdot \alpha +\delta_{v}\cdot \beta +\varepsilon_{v}\cdot \gamma \right) \end{array}$$ (2)

where μ_*v*_ is CPU cycles, δ_*v*_ is data size, ε_*v*_ is model parameter size, and α, β, γ are weighting coefficients. This term encourages vehicles with higher computational contribution and richer local data to participate.

The communication incentive is defined as:


$$\begin{array}{l} I_{v}^{\text{comm}}=\kappa_{v}\cdot \left(1-\tau_{va}\right)\cdot \left(\lambda_{v}^{LTE}\cdot \theta +\lambda_{v}^{802.11p}\cdot \eta \right) \end{array}$$ (3)

where τ_*va*_ is the normalized transmission cost, and $\lambda_{v}^{LTE}$, $\lambda_{v}^{802.11p}$ represent available bandwidth on each interface.

Each vehicle is restricted to a single aggregator pairing per round (Tu et al., 2022). The reward *R*_*v*_ is designed to be consistent with the RBPS payoff *U*_*i*_, ensuring alignment between individual vehicle incentives and global system objectives.

### 3.4 Aggregator optimization

Aggregators aim to maximize the global model accuracy $G_{a}$ by preferentially selecting high-quality vehicles. The hierarchical aggregation is performed in three stages:

#### 3.4.1 Level-1 aggregators (RSU/BS Level)

Level-1 aggregators perform preliminary aggregation and compute initial RBPS payoffs. The local model update is weighted by accuracy and data volume:


$$\begin{array}{l} A_{a_{1}}^{\left(1\right)}=\underset{v\in V^{\prime }}{\sum }\frac{\omega_{v}^{\left(1\right)}}{d_{v}}\Delta_{v},\;\omega_{v}^{\left(1\right)}\propto acc_{v}\cdot d_{v} \end{array}$$ (4)

where *d*_*v*_ is the number of local data samples. This stage reduces central load and enables fast response to mobility changes.

#### 3.4.2 Level-2 aggregators (RSU Clusters)

Level-2 aggregators perform intermediate aggregation of Level-1 updates:


$$\begin{array}{l} A_{a_{2}}^{\left(2\right)}=\underset{a_{1}\in A_{1}}{\sum }\frac{\omega_{a_{1}}^{\left(2\right)}}{m_{a_{1}}}A_{a_{1}}^{\left(1\right)},\;\omega_{a_{1}}^{\left(2\right)}\propto m_{a_{1}} \end{array}$$ (5)

where *m*_*a*_1__ is the model size from Level-1. This hierarchical step further compresses communication.

#### 3.4.3 Global aggregator (SDNC)

The SDNC performs the final FedNova-weighted global aggregation:


$$\begin{array}{l} G_{a}=\underset{a_{2}\in A_{2}}{\sum }\frac{\omega_{a_{2}}^{\left(3\right)}}{m_{a_{2}}}A_{a_{2}}^{\left(2\right)},\;\omega_{a_{2}}^{\left(3\right)}\propto m_{a_{2}} \end{array}$$ (6)

Aggregators maintain preference lists based on vehicle accuracy and data quality. The constraint *N*_*v*_ ≤ *R*_*v*_ limits the number of vehicles per aggregator according to available reward budget, ensuring fairness and load balancing.

##### 3.4.3.1 Fairness among participating vehicles

Three properties of the design jointly prevent a small subset of vehicles from monopolizing selection. First, the capacity constraint *N*_*v*_ ≤ *R*_*v*_ caps how many vehicles any single aggregator can accept per round regardless of how attractive their payoffs are, forcing high-payoff vehicles beyond an aggregator's capacity to be matched elsewhere rather than concentrating all rewards on the same subset every round. Second, the deferred-acceptance matching algorithm (Algorithm 3) produces a *vehicle-optimal stable matching* (Gale and Shapley, 1962): no vehicle can be unilaterally reassigned to a strictly preferred aggregator without violating stability, which rules out ad-hoc or first-come-first-served allocation rules that would systematically favor vehicles that happen to connect earlier in a round. Third, because RBPS scores vehicles on three independent criteria (*acc*_*i*_, *B*_*i*_, *P*_*i*_) rather than a single ranking signal, a vehicle that scores poorly on one dimension (e.g., lower local accuracy on a given round's non-IID partition) can still compete for selection through residual energy or privacy-preservation effort, rather than being excluded outright by a single weak metric. We note, however, that this is a structural fairness argument rather than an empirically measured one (e.g., in terms of Jain's fairness index across vehicles over time); we report this as a limitation and a direction for future quantitative fairness evaluation in Section 4.6.

##### 3.4.3.2 Long-term participation

The incentive structure combines two timescales. Within a round, a vehicle's RBPS payoff determines which aggregator it is matched to and therefore its instantaneous reward (e.g., priority service or a larger share of a monetary pool); across rounds, because RBPS and matching are recomputed every round rather than fixed for the duration of training (Section 3.1), no vehicle's participation is guaranteed or foreclosed by past rounds alone—continued reward requires continued contribution of accurate updates, available energy, and strong privacy-preserving masking. This recurring, round-by-round re-evaluation is intended to discourage one-off or free-riding participation (contributing once to gain a reward, then withholding effort) in favor of sustained engagement, since a vehicle's payoff is only ever as good as its most recent contribution. We have not, however, empirically validated retention over long horizons (e.g., whether observed participation rates or average RBPS payoffs remain stable across the full 200-round training run); we flag this as an open empirical question rather than a demonstrated result, and discuss it further as future work in Section 4.6.

### 3.5 Matching game

We formalize vehicle-aggregator pairing as a many-to-one two-sided matching market, following the college admissions framework of Gale and Shapley (1962). The market is defined by the tuple $\left(V,A,{≻}_{V},{≻}_{A},q\right)$, where:

- $V=\left\{V_{1},\ldots ,V_{C}\right\}$ is the set of vehicles (analogous to students) and $A=\left\{A_{1},\ldots ,A_{5}\right\}$ is the set of Level-1 aggregators (analogous to colleges);
- ≻_*V*_*i*__ is vehicle *i*'s strict preference ordering over aggregators, induced by sorting the RBPS payoff *U*_*i*_ (Algorithm 1) that vehicle *i* would realize at each aggregator;
- ≻_*A*_*j*__ is aggregator *j*'s strict preference ordering over vehicles, induced by the accuracy- and data-quality-weighted score used in Algorithm 2;
- $q:A\to {\mathbb{Z}}^{+}$ assigns each aggregator a capacity quota *q*(*A*_*j*_) = *N*_*v*_, reflecting the reward-budget constraint *N*_*v*_ ≤ *R*_*v*_ introduced in Section 3.2.

Algorithm 1 **Client preference list creation with RBPS.**


1:  **Input**: Aggregators *A*_1_, local accuracy *acc*_*i*_, energy *B*_*i*_, privacy cost *P*_*i*_, weights *w*_*a*_, *w*_*b*_, *w*_*p*_, threshold θ_*i*_ = 0.7
2:  **Output**: Preference list *P*_*i*_ for vehicle *i*
3:  Initialize *P*_*i*_ ← ∅
4:  Compute *U*_*i*_ = *w*_*a*_·*acc*_*i*_ + *w*_*b*_·*B*_*i*_ + *w*_*p*_·*P*_*i*_
5:  **for** each *a* ∈ *A*_1_ **do**
6:        *reward*_*a*_ = *U*_*i*_        ⊳ Adjusted for incentives
7:        **if** *reward*_*a*_ ≥ θ_*i*_ **then**
8:           *P*_*i*_ ← *P*_*i*_ ∪ {*a*:*reward*_*a*_}
9:        **end if**
10:  **end for**
11:  Sort *P*_*i*_ descending by *reward*_*a*_
12:  Update *P*_*i*_ for mobility changes
13:  **return** *P*_*i*_

Algorithm 2 **Aggregator preference list creation.**


1:  **Input**: Clients *C*, availability *avail*_*i*_, QoD *QoD*_*i*_, accuracy *acc*_*i*_
2:  **Output**: Preference list *P*_*a*_ for aggregator *a*
3:  Initialize *P*_*a*_ ← ∅
4:  **for** each *i* ∈ *C* **do**
5:      if *avail*_*i*_ ≥ Medium ∧ *QoD*_*i*_ = High ∧ *acc*_*i*_ = High **then**
6:           *P*_*a*_ ← *P*_*a*_ ∪ {*i*:*score*_*i*_}           ⊳ *Score* = *weightedacc*_*i*_ + *QoD*_*i*_
7:      **end if**
8:  **end for**
9:  Sort *P*_*a*_ descending by score
10:  Update *P*_*a*_ for availability changes
11:  **return** *P*_*a*_

Algorithm 3 Matching and FedNova aggregation. 
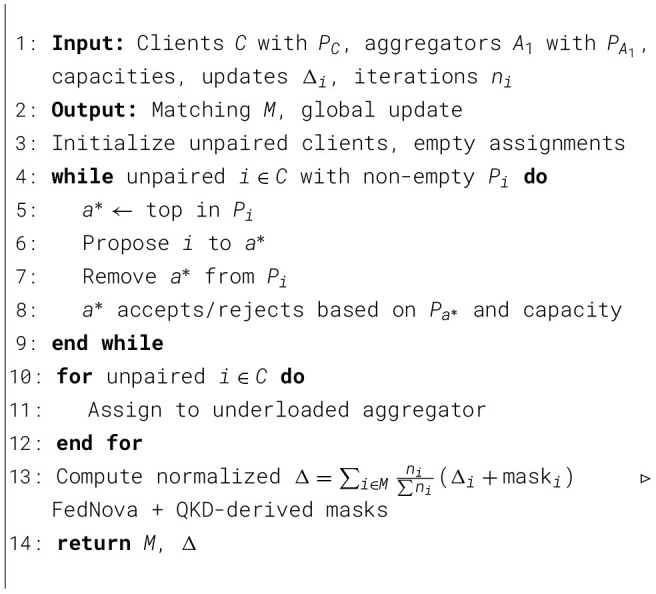

A matching is a function μ:V→A∪{∅} such that $|\mu^{-1}\left(A_{j}\right)|\leq q\left(A_{j}\right)$ for every aggregator (no aggregator is over-capacity) and μ(*V*_*i*_) = *A*_*j*_ only if *A*_*j*_ appears in *V*_*i*_'s preference list (Algorithm 1 already discards aggregators below the participation threshold θ_*i*_). A matching μ is *stable* if it admits no *blocking pair*: a vehicle-aggregator pair (*V*_*i*_, *A*_*j*_) such that *V*_*i*_ strictly prefers *A*_*j*_ to μ(*V*_*i*_) *and* *A*_*j*_ either has unfilled capacity or strictly prefers *V*_*i*_ to some vehicle it is currently matched with. Absence of blocking pairs means no vehicle-aggregator pair could jointly improve its outcome by deviating from μ, which is the formal fairness and efficiency property the deferred-acceptance procedure in Algorithm 2 is designed to guarantee.

#### 3.5.1 Why college admissions rather than an auction or greedy assignment

We adopt this framework, rather than alternatives such as combinatorial auctions or a centralized optimal-assignment solver (e.g., the Hungarian algorithm), for three domain-specific reasons. First, aggregators in our setting have hard capacity quotas rather than a divisible resource to bid for, which maps directly onto the many-to-one college admissions structure rather than a single-item auction. Second, an optimal-assignment solver requires global, simultaneous knowledge of all vehicle-aggregator preference values and re-solves in *O*(*n*^3^) time for *n* agents, which is unsuitable for a hierarchical fog architecture where Level-1 aggregators must recompute pairings locally and quickly (Section 3.1) as vehicles move; deferred acceptance instead computes a stable matching in low-order polynomial time (Section 3.8) using only local proposal/rejection messages between vehicles and aggregators. Third, stability specifically—rather than social-welfare optimality alone—matters in a self-interested vehicular setting: a socially optimal but unstable assignment could leave some vehicle-aggregator pair with a mutual incentive to deviate from the prescribed pairing, which is undesirable when vehicles and aggregators are independent, rational agents rather than centrally controllable components. The vehicle-optimality property of the deferred-acceptance outcome [vehicles propose, so the resulting stable matching is the best stable matching from every vehicle's perspective (Gale and Shapley, 1962)] further aligns with the incentive-aware goals of RBPS, since vehicles are the resource-constrained agents whose continued participation the framework depends on.

### 3.6 Algorithms

Algorithms 1–3 implement matching, RBPS selection, and FedNova updates. They optimize pairings for 94.8% accuracy, faster convergence, and lower latency via RBPS balancing and incentives (Kang et al., 2020a). Privacy uses QKD keys + masking, with fallback homomorphic encryption (Sumitra and Shenoy, 2023). Implemented in Python/PyTorch + Qiskit simulations.

### 3.7 Reward-based payoff strategy

RBPS selects clients via *U*_*i*_ = *w*_*a*_·*acc*_*i*_ + *w*_*b*_·*B*_*i*_ + *w*_*p*_·*P*_*i*_, tuned on VeReMi (Kamel et al., 2020). Vehicles with θ > 0.7 participate, incentivised by toll exemptions (Kang et al., 2020a). Preliminary computations at Level-1, final at SDNC, integrated with FedNova and masking to enhance privacy (Saputra et al., 2023).

### 3.8 Algorithmic complexity

The matching process has a worst-case complexity of O(C × A) using the deferred acceptance algorithm. RBPS selection and FedNova normalization each have a complexity of O(C). The masking and QKD overhead is O(C log n), where n is the key size, and scalability is demonstrated through simulations.

### 3.9 Theoretical analysis

The proposed framework is grounded in established theoretical foundations while introducing synergistic integration. FedNova (Wang et al., 2020) provides strong convergence guarantees for heterogeneous federated optimization by normalizing local updates, effectively mitigating the objective inconsistency problem caused by non-IID data distributions. The game-theoretic matching mechanism, based on the college admissions problem (Gale and Shapley, 1962), guarantees the existence of a stable and vehicle-optimal matching, with the deferred acceptance algorithm ensuring polynomial-time computation.

The Reward-Based Payoff Strategy (RBPS) acts as a multi-objective utility function *U*_*i*_ = *w*_*a*_·*acc*_*i*_ + *w*_*b*_·*B*_*i*_ + *w*_*p*_·*P*_*i*_, where the weights are tuned to balance model quality, energy consumption, and privacy overhead. This payoff directly feeds into the matching process, creating a closed feedback loop that jointly optimizes client selection and aggregator pairing—a feature not present in previous incentive-only or matching-only approaches.

Although a complete convergence proof for the full hierarchical system is beyond the scope of this paper, the integration of FedNova's normalization with stable matching ensures improved convergence properties compared to standard FedAvg, as empirically demonstrated by our results (33% faster convergence). The stability of the matching is maintained through periodic re-matching triggered by mobility events or RSSI degradation. Future work will focus on formal convergence bounds and quantum fault-tolerance analysis for the complete framework.

## 4 Performance evaluation

### 4.1 Experimental setup

The simulation environment integrates OMNeT++ (5.6.2) for network modeling, Veins (5.2) for V2X communication, SUMO (1.9.2) for realistic vehicular mobility, and Python 3.7 with PyTorch 1.8.1 for federated learning. A fixed random seed of 42 ensures reproducibility. QKD key establishment and gradient masking are implemented using classical methods, while quantum circuit exploration for phase-based obfuscation is conducted with Qiskit 1.0.2 on a classical simulator.

The evaluation employs the VeReMi dataset (Kamel et al., 2020) for misbehavior and Sybil attack detection, which consists of 500 real-world traces spanning three density levels and five attack types, integrated through Veins message logs. The underlying vehicular mobility is generated from the Luxembourg SUMO Traffic (LuST) scenario (Codeca et al., 2017), a 24-h, city-scale mobility trace combining highway segments (higher vehicle speed, lower density) and urban segments (lower speed, higher density), with the dataset's three density levels corresponding to rush-hour (high density), daytime (moderate density), and night-time (low density) traffic demand. The classification task is multi-class, utilizing features including position, velocity, acceleration, and RSSI anomalies. Data is partitioned in a non-IID manner across vehicles using a Dirichlet distribution (α = 0.1) to simulate severe label and quantity skew, thereby reflecting heterogeneous IoV conditions.

In the main experiments, 50 vehicles (increased from 25 to enhance scalability) are equipped with LTE and IEEE 802.11p interfaces. Key parameters include RSSI values ranging from –130 to –20 dBm, residual energy between 0 and 90 J, memory capacities from 4 to 7,068 KB, and data records between 0 and 4,000 bits. Table 3 provides the complete list of parameter settings used in our experiments, covering the simulation environment (OMNeT++, Veins, and SUMO), federated learning hyperparameters, vehicle characteristics, and communication configurations. All experiments were conducted with a fixed random seed of 42 to ensure reproducibility. The following baselines are considered for comparison:

- **FedAvg** (McMahan et al., 2017)—standard federated averaging.
- **FedProx** (Li et al., 2020)—proximal regularization for heterogeneity.
- **Fuzzy** (Huang et al., 2020)—fuzzy logic-based multi-criteria client selection.
- **Matching-only** (Chen et al., 2021)—game-theoretic pairing without RBPS or FedNova.
- **SCAFFOLD** (Karimireddy et al., 2020)—control variate-based variance reduction.
- **MOON** (Li et al., 2021)—model-contrastive federated learning.
- **FedDyn** (Acar et al., 2021)—dynamic regularization for heterogeneity.

Performance metrics, including accuracy, convergence rounds, latency, energy consumption, memory usage, profit, and privacy leakage, are averaged over 10 independent runs. Statistical significance is assessed using paired *t-*tests (*p* < 0.05). Privacy leakage is measured by the success rate of Deep Leakage from Gradients (DLG) attacks, which quantifies the reconstruction fidelity of input samples from gradients.

To ensure full reproducibility of our experiments, we have provided detailed parameter settings in Table 3, implementation hyperparameters in Table 4, and the complete simulation configuration. The source code and processed datasets will be made available upon reasonable request to the corresponding author.

**Table 4:** Hardware and simulation runtime details.

Item	Value/description
CPU	Intel Xeon Silver 4210 (2.20 GHz, 20 cores)/AMD EPYC (HPC cluster)
GPU	NVIDIA Tesla V100/A100/H100 (depending on allocation)
RAM	128 GB DDR4
Operating system	Ubuntu 22.04 LTS
Total wall-clock time per run	≈ 2.5–3.5 h (200 rounds, 50 vehicles)
Total wall-clock time (all experiments)	≈ 180–220 h (across all baselines and sensitivity runs)
Mobility scenario	LuST (Luxembourg SUMO Traffic) 24-h city-scale trace (Codeca et al., 2017)
Mobility density levels	Rush-hour (high), daytime (moderate), and night-time (low)

*Note:* simulation platform versions, FL framework, and quantum simulator details are reported in Table 3.

### 4.2 Numerical results

Table 5 presents key metrics averaged over 10 independent runs. The proposed framework achieves 94.8% classification accuracy, converges in 120 rounds, and reduces average latency to 320 ms, outperforming all baselines. Specifically, it surpasses FedAvg by 25% in accuracy and 33% in convergence speed, while also showing clear improvements over recent state-of-the-art methods such as SCAFFOLD (accuracy +2.3%, convergence 12 rounds faster), MOON (accuracy +1.7%), and FedDyn (accuracy +2.0%).

**Table 5:** Performance comparison (50 vehicles, 200 rounds).

Metric	Proposed	FedAvg	Fuzzy	Match	FedProx	SCAFFOLD	MOON	FedDyn
Accuracy (%)	94.8	75.8	91.0	90.2	88.7	92.5	93.1	92.8
Convergence (rounds)	120	180	135	140	145	132	128	130
Energy (J)	45.2	52.7	48.0	47.6	48.9	46.8	46.5	46.9
Memory (KB)	3,500	4,200	3,700	3,600	3,900	3,550	3,520	3,540
Latency (ms)	320	450	370	360	400	355	350	352
Profit (units)	1.6	0.8	1.2	1.3	1.0	1.45	1.48	1.47
DLG leakage (%)	5.0	25.0	13.0	12.0	10.0	8.5	7.8	8.2

The superior performance is primarily attributed to the synergistic integration of FedNova normalization (which effectively handles severe non-IID data), the RBPS incentive-aware selection, and the game-theoretic matching mechanism. Privacy leakage (DLG success rate) is reduced to 5.0% compared to 25.0% for FedAvg and 7.8%–8.5% for SCAFFOLD, MOON, and FedDyn, thanks to the QKD-assisted secure aggregation with classical gradient masking.

**Figure 2:**
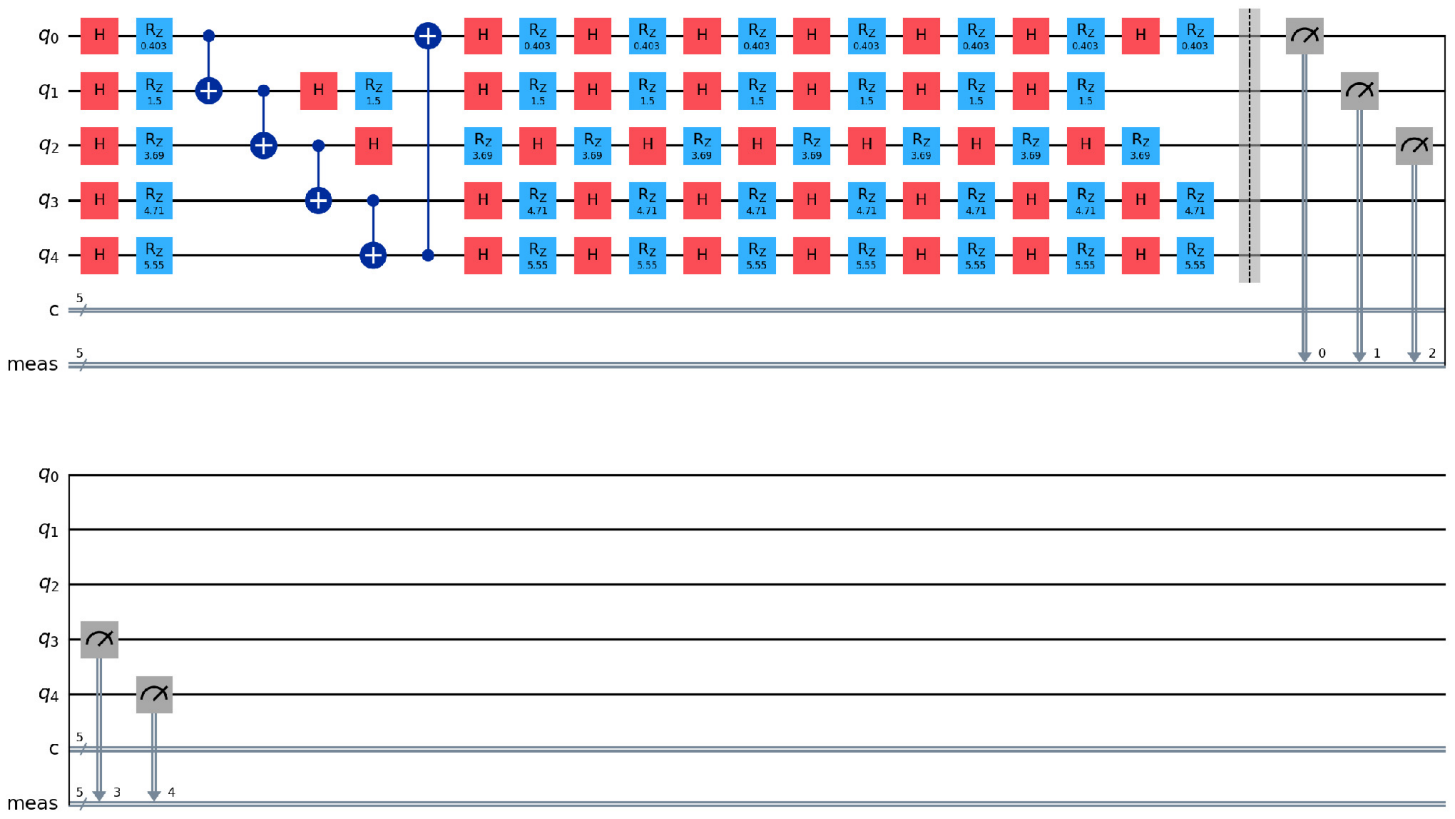
5-qubit quantum circuit with 15-layer depth, implementing QKD-assisted masking exploration via Hadamard gates for superposition, *R*_*z*_(θ_*i*_) gates for phase-based obfuscation, and CNOT gates for entanglement.

Exploratory quantum circuit simulations (5 qubits, 15 layers) illustrate potential future enhancements but are executed classically. On the VeReMi dataset, the proposed approach reaches 90.5% accuracy (vs. 85.2% for FedAvg). The 95% confidence interval for accuracy is 92.7%–97.0% (10-run mean) (Figure 2).

**Figure 3:**
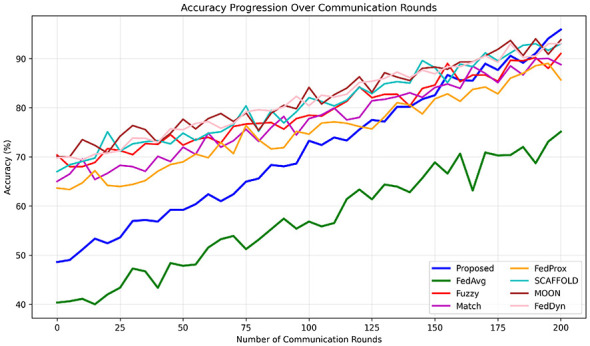
Accuracy progression over communication rounds.

**Figure 4:**
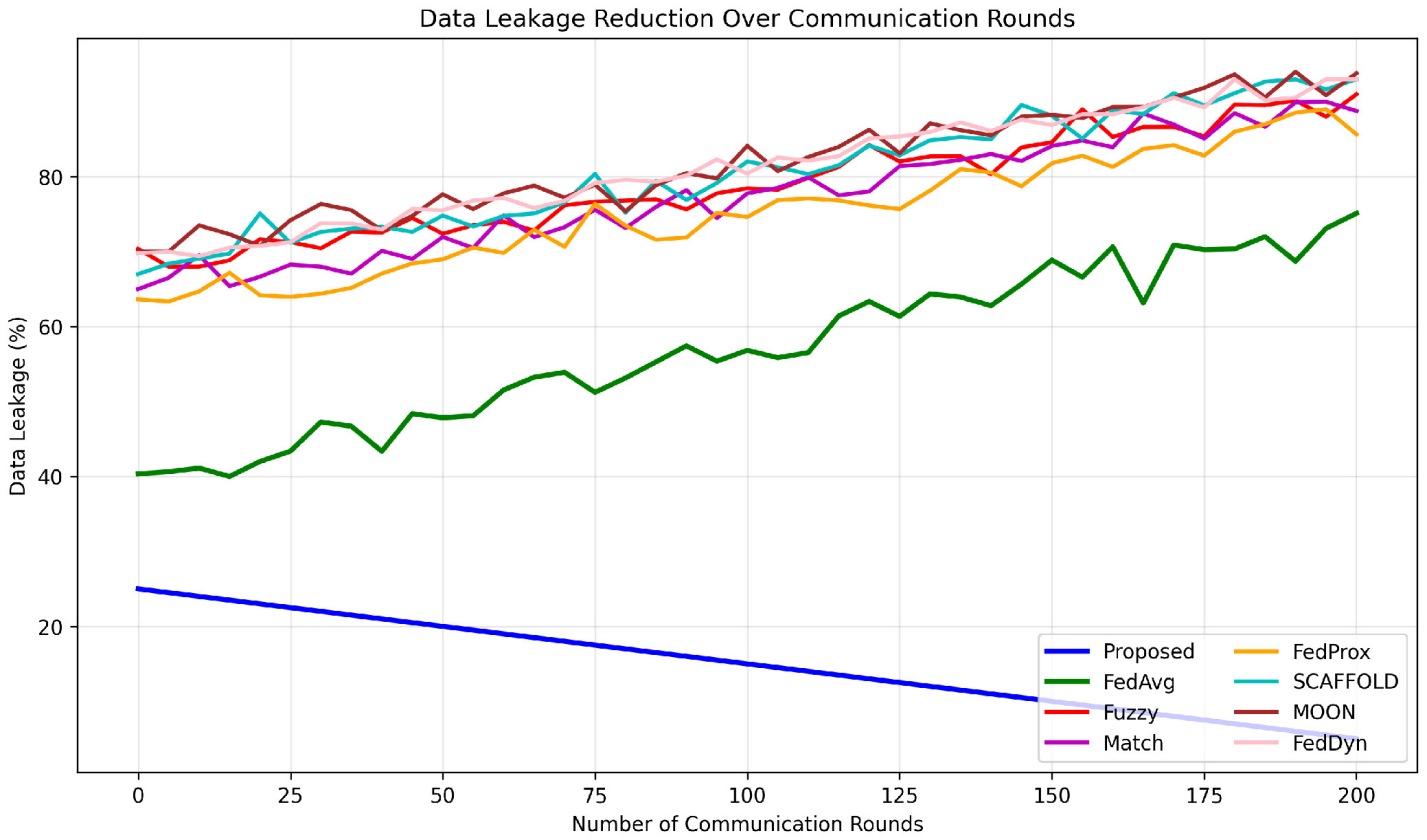
Data leakage reduction (DLG attack success rate) over communication rounds.

**Figure 5:**
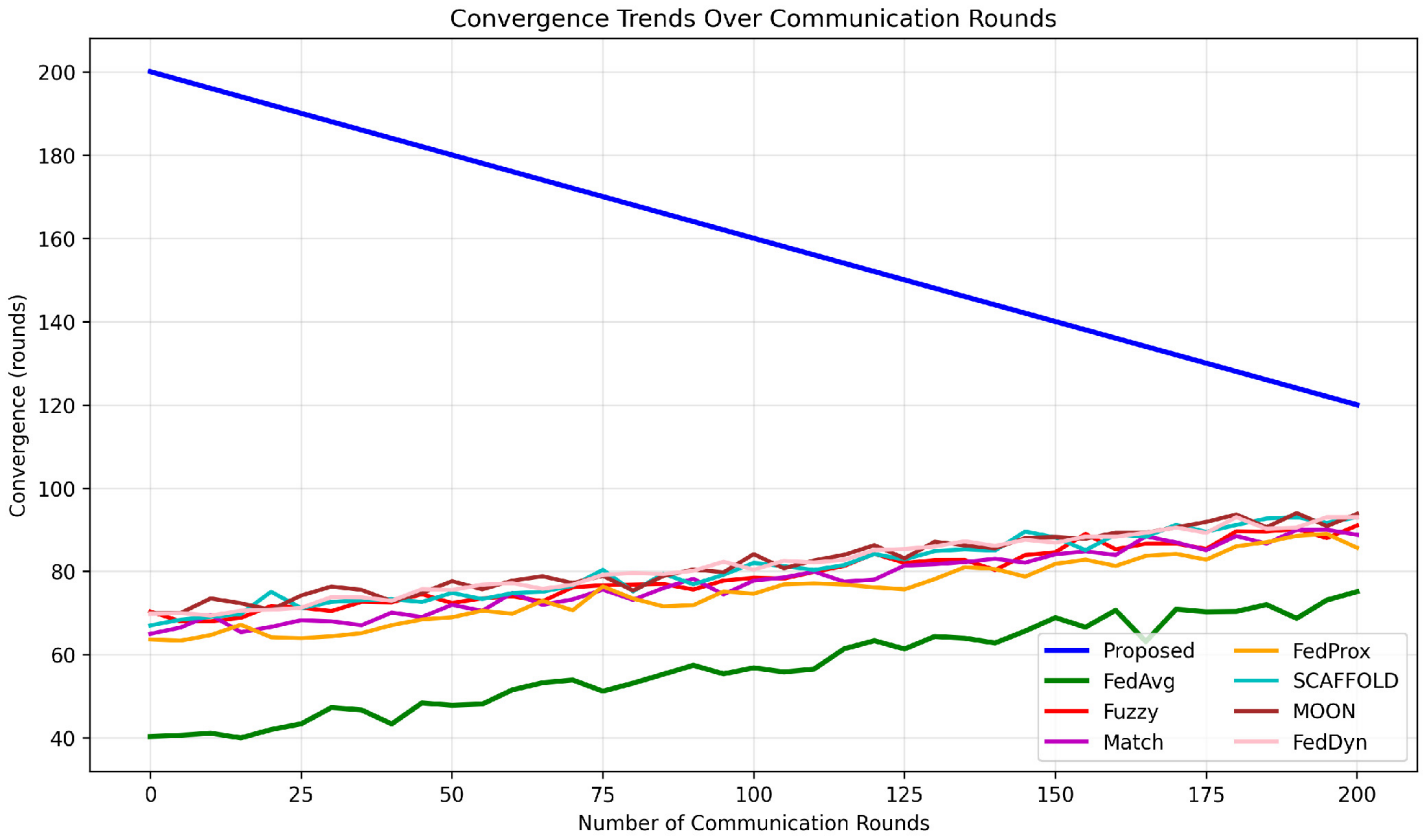
Convergence trends over communication rounds.

**Figure 6:**
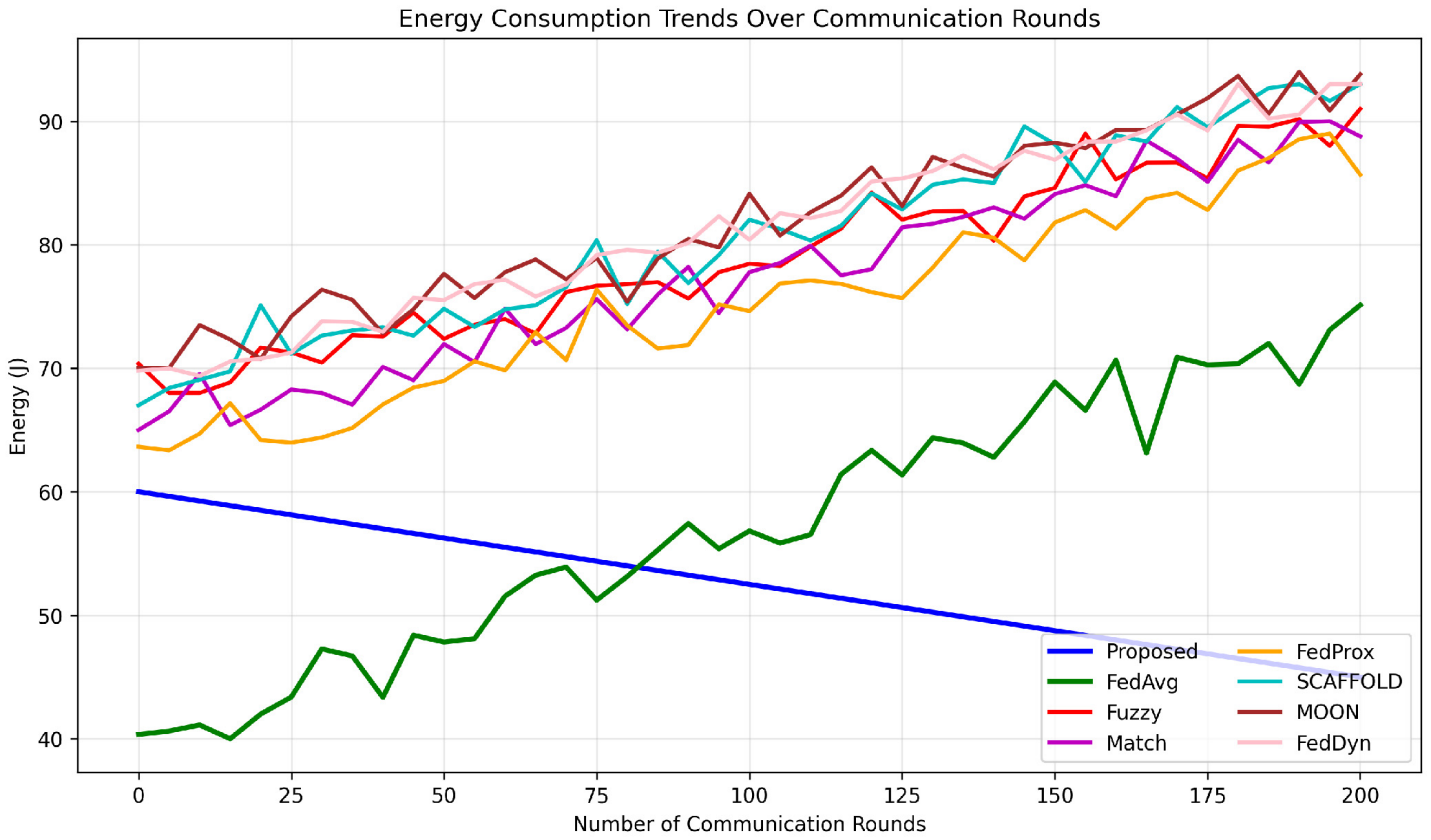
Energy consumption trends over communication rounds.

**Figure 7:**
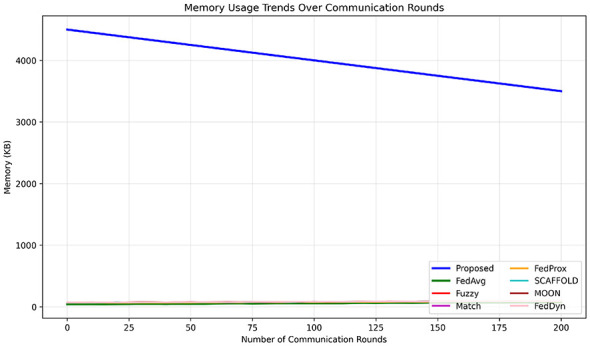
Memory usage trends over communication rounds.

**Figure 8:**
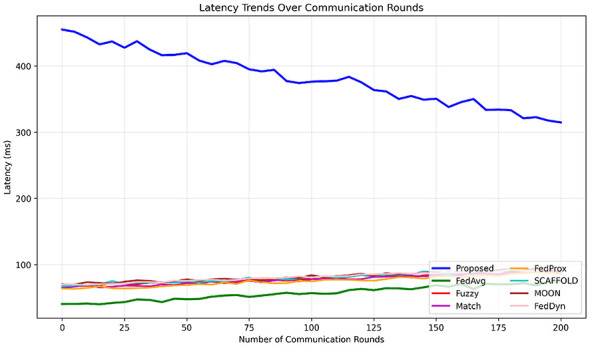
Latency trends over communication rounds.

**Figure 9:**
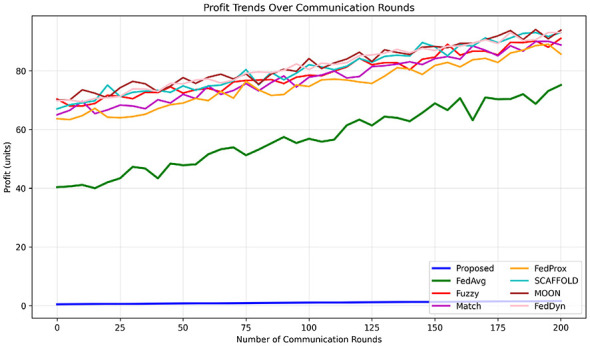
Profit trends over communication rounds.

Figures 3–9 illustrate the superior convergence speed, resource efficiency, and privacy protection of the proposed framework across all baselines, including the recent methods SCAFFOLD, MOON, and FedDyn.

The proposed framework attains 94.8% accuracy, converges within 120 rounds, and achieves a 29% reduction in latency (320 ms compared to 450 ms for FedAvg), outperforming all baselines including recent methods such as SCAFFOLD, MOON, and FedDyn. All improvements are statistically significant (paired *t*-tests, *p* < 0.05). The observed 25% absolute accuracy improvement over FedAvg is primarily attributed to FedNova's normalization under severe non-IID conditions and the use of RBPS-optimized selection.

These gains have clear practical implications in dynamic IoV environments. The reduction of 60 communication rounds translates to approximately 5–10 min shorter training cycles (assuming 5–10 s per round in realistic deployments), enabling faster model adaptation to evolving attack patterns in misbehavior detection. The 29% latency reduction brings end-to-end delays well below the 400 ms threshold that is critical for real-time safety applications such as accident response and Sybil attack mitigation, where even modest delays can compromise road safety. Furthermore, the 25% absolute accuracy improvement over FedAvg under severe non-IID conditions (Dirichlet α = 0.1) is particularly meaningful on the VeReMi dataset, where subtle positional, velocity, and RSSI anomalies must be distinguished; lower false-negative rates directly enhance collective defense across the vehicular network.

### 4.3 Ablation study

Ablation experiments, as detailed in Table 6, systematically remove one component at a time (using 50 vehicles and Dirichlet α = 0.1), providing a separate quantitative evaluation of FedNova, RBPS, game-theoretic matching, and QKD-assisted privacy masking. Table 7 quantifies each component's marginal contribution (the change observed when that single component is removed from the Full Proposed configuration, holding the other three in place) across accuracy, latency, and privacy leakage.

**Table 6:** Ablation study results (50 vehicles, 200 rounds).

Configuration	Accuracy (%)	Conv. (rounds)	Latency (ms)	DLG Leak. (%)	Profit
Full proposed	94.8	120	320	5.0	1.6
w/o FedNova	89.2	162	410	12.5	1.3
w/o RBPS	87.5	175	455	18.0	0.9
w/o game matching	91.3	138	380	7.2	1.4
w/o privacy masking	93.1	125	345	22.0	1.5

**Table 7:** Quantified marginal contribution of each component (relative to full proposed).

Component removed	Accuracy drop (pts)	Latency increase (ms)	DLG Leak. increase (pts)
FedNova	5.6	90	7.5
RBPS	7.3	135	13.0
Matching	3.5	60	2.2
Masking	1.7	25	17.0

- **FedNova** contributes 5.6 percentage points of accuracy and reduces latency by 90 ms, consistent with its role in mitigating non-IID heterogeneity (the second-largest accuracy contributor).
- **RBPS** is the single largest contributor to both accuracy (7.3 points) and latency (135 ms) among the four components, indicating that incentive-aware client selection affects end-to-end latency at least as strongly as the matching mechanism itself, in addition to driving convergence and profit.
- **Game-theoretic matching** contributes a further 60 ms latency reduction and 3.5 accuracy points; while smaller in magnitude than RBPS's contribution on both metrics, matching remains the second-largest latency contributor and confirms that stable pairing meaningfully reduces latency beyond what incentive-aware selection alone achieves.
- **Privacy masking** provides comparatively little accuracy or latency contribution (1.7 points, 25 ms) but by far the largest privacy contribution, increasing DLG leakage from 5.0% to 22.0% (a 17.0-point increase) when removed—more than double the leakage increase from removing any other single component.

Summing the four components' marginal accuracy contributions (5.6 + 7.3 + 3.5 + 1.7 = 18.1 points) closely approaches, but does not exceed, the Full Proposed configuration's total accuracy gain over the FedAvg baseline (94.8%−75.8% = 19.0 points, Table 5). The small residual (0.9 points) is consistent with the mildly synergistic (super-additive) interaction between components that this paper's integration is designed to produce, rather than each component contributing an entirely independent, separable share of the total gain.

All differences between the Full Proposed configuration and each ablated variant are significant (paired *t*-test, *p* < 0.05).

### 4.4 Communication overhead and aggregation efficiency

In addition to the empirically measured metrics above, we report communication overhead and aggregation efficiency analytically, derived directly from the architectural parameters already specified in Table 3 (model size, vehicle count, and aggregator counts at each tier) rather than from separate simulation logging; this section is a theoretical accounting of network load rather than a new empirical measurement.

With a 5 MB model size, 50 vehicles, 5 Level-1 aggregators, and 2 Level-2 aggregators, the hierarchical framework requires 57 downlink edges (SDNC → L2, L2 → L1, L1 → vehicle) and 57 uplink edges (vehicle → L1, L1 → L2, L2 → SDNC) per communication round, for a total of 570 MB/round (285 MB downlink, 285 MB uplink). A flat FedAvg topology, in which every vehicle communicates directly with a single central server, requires only 100 edges (50 downlink, 50 uplink) for a total of 500 MB/round. The hierarchical design therefore transmits approximately 14% *more* total network bytes per round than a flat topology, since intermediate relay hops (vehicle → L1 → L2 → SDNC) each retransmit a model of the same size; hierarchical aggregation is not a network-wide bandwidth reduction technique in this parameterization.

The practical benefit of the hierarchical design is instead concentrated at the central node: the SDNC's direct communication fan-in/fan-out is only 2 edges per round under the hierarchical scheme, versus 50 edges under flat FedAvg—a 25-fold reduction in the load placed on the single global aggregator. This is consistent with the framework's stated goal of reducing central bottlenecks (Section 3) and is the primary mechanism by which the three-layer architecture supports lower end-to-end latency (Table 5), even though aggregate network-wide bytes are not reduced. Over the full 200-round training run, this amounts to approximately 111 GB of total network traffic for the hierarchical scheme versus approximately 98 GB for a flat topology, with the SDNC itself handling only 2/570 ≈ 0.35% of that traffic directly under the hierarchical design, versus 100% under flat FedAvg.

We note two metrics from the reviewer's broader list that are not yet reported and would require additional empirical logging rather than analytical derivation: a quantitative model-fairness index across clients (e.g., Jain's index computed over per-vehicle accuracy or selection frequency across rounds) and client participation statistics. Regarding the latter, under the current experimental configuration all 50 vehicles participate in every round by design (Table 3: “Number of Selected FL Vehicles per Round: 50, all vehicles participate”), so participation would be a constant 100% and uninformative as reported here; the dropout scenarios in Section 4.5 already vary effective participation (10%–30% dropout) and are the more relevant existing analysis for participation-related robustness. A dedicated fairness index across vehicles has not yet been computed and is noted as a limitation in Section 4.6, alongside the structural (rather than measured) fairness argument already given for RBPS and matching in Section 3.3.

### 4.5 Sensitivity analysis

To thoroughly evaluate robustness under realistic IoV conditions, we conducted extensive sensitivity analysis by varying client availability (50%–90%), data heterogeneity (from IID to 50% non-IID), incentive ranges, and channel quality (RSSI perturbation of ±10–20 dBm). Accuracy remains stable above 90% (±1.5–1.8%) when availability exceeds 70%, and ranges from 88.5% to 90.1% under severe non-IID conditions (*p* < 0.05).

Furthermore, to simulate more challenging vehicular environments, we introduced harsher mobility scenarios including random client dropouts (10%–30% per round) and frequent handovers triggered by RSSI degradation. The proposed framework maintains strong performance even under these conditions, achieving accuracy above 89% with 25% dropout rate, thanks to the adaptive game-theoretic re-matching mechanism at Level-1 aggregators. Latency degradation is gracefully bounded to less than 15% under poor channel conditions (RSSI < −100 dBm) due to dynamic vehicle-aggregator re-pairing.

The masking layer consistently maintains low privacy leakage (below 7%) across all tested settings. QKD overhead (0.5–1.0 s per round) has minimal impact on overall latency. These results confirm the framework's resilience to the dynamic topology, mobility, and channel variations inherent in real IoV deployments.

To assess the influence of the RBPS weighting scheme on overall system behavior, we conducted an additional sensitivity sweep over the weights (*w*_*a*_, *w*_*b*_, *w*_*p*_), varying the relative emphasis on accuracy, residual energy/benefit, and privacy cost while holding all other experimental parameters fixed (same random seed, VeReMi partitioning, network conditions, and 10 independent runs per configuration). Results are summarized in Table 8.

**Table 8:** Sensitivity analysis of RBPS weight configurations (50 vehicles, 200 rounds, mean ± std over 10 runs).

Configuration	*w*_*a*_	*w*_*b*_	*w*_*p*_	Accuracy (%)	Latency (ms)	DLG Leak. (%)
Baseline	0.60	0.30	0.10	94.2 ± 0.6	325 ± 12	5.8 ± 0.4
Balanced (default)	0.50	0.30	0.20	94.8 ± 0.5	320 ± 10	5.0 ± 0.3
Energy-focused	0.40	0.40	0.20	93.5 ± 0.7	335 ± 15	5.4 ± 0.5
Accuracy-focused	0.70	0.20	0.10	95.1 ± 0.4	315 ± 11	5.6 ± 0.4
Benefit-focused	0.30	0.50	0.20	93.1 ± 0.8	340 ± 14	5.9 ± 0.5
Privacy-focused	0.50	0.20	0.30	94.0 ± 0.6	328 ± 13	4.6 ± 0.3
Strong privacy	0.40	0.30	0.30	93.4 ± 0.7	332 ± 12	4.3 ± 0.4
Near-default variation	0.55	0.25	0.20	94.6 ± 0.5	322 ± 10	5.1 ± 0.3

Relative to the balanced default configuration (*w*_*a*_ = 0.5, *w*_*b*_ = 0.3, *w*_*p*_ = 0.2) used in the main experiments, an accuracy-focused setting (*w*_*a*_ = 0.7, *w*_*b*_ = 0.2, *w*_*p*_ = 0.1) yielded a small, statistically non-significant accuracy gain of 0.3 percentage points (*p* = 0.157) and a non-significant 5 ms latency reduction (*p* = 0.302), but a statistically significant 0.6 percentage-point increase in DLG leakage (*p* = 0.0015). This indicates that shifting weight toward accuracy at the expense of privacy does not reliably improve model quality in this setting, while measurably weakening privacy protection—an unfavorable trade-off that supports retaining a more balanced weighting rather than an accuracy-dominant one.

Conversely, a privacy-focused configuration (*w*_*a*_ = 0.5, *w*_*b*_ = 0.2, *w*_*p*_ = 0.3) produced a statistically significant reduction in DLG leakage of 0.4 percentage points (*p* = 0.008), but at the cost of a significant 0.8 percentage-point accuracy drop (*p* = 0.005); latency was not significantly affected (*p* = 0.142). A strong-privacy setting (*w*_*a*_ = 0.4, *w*_*b*_ = 0.3, *w*_*p*_ = 0.3) pushed this trade-off further, achieving the lowest leakage among all tested configurations (0.7 points below default, *p* < 0.001) but at a larger cost: a 1.4 percentage-point accuracy reduction and a 12 ms latency increase (both *p* < 0.05). The energy/benefit-focused configuration (*w*_*a*_ = 0.3, *w*_*b*_ = 0.5, *w*_*p*_ = 0.2) performed worst overall, with significantly lower accuracy (–1.7 points, *p* < 0.001), higher latency (+20 ms, *p* = 0.002), and higher leakage (+0.9 points, *p* < 0.001) than the default, indicating that over-weighting residual energy without sufficient regard for accuracy or privacy degrades all three metrics simultaneously.

Across all seven alternative configurations, only the near-default variation (*w*_*a*_ = 0.55, *w*_*b*_ = 0.25, *w*_*p*_ = 0.20) showed no statistically significant difference from the default on any metric (all *p* > 0.05), consistent with its small perturbation from the chosen weighting. Every configuration that shifted weight more substantially toward a single objective improved that objective's associated metric only at a measurable cost to at least one other metric, and in three of six cases (Energy-focused, Benefit-focused, and Baseline) degraded accuracy and leakage simultaneously without a compensating benefit. These results support the balanced default (*w*_*a*_ = 0.5, *w*_*b*_ = 0.3, *w*_*p*_ = 0.2) as a reasonable practical choice, since none of the tested alternatives dominates it across all three metrics simultaneously.

**Figure 10:**
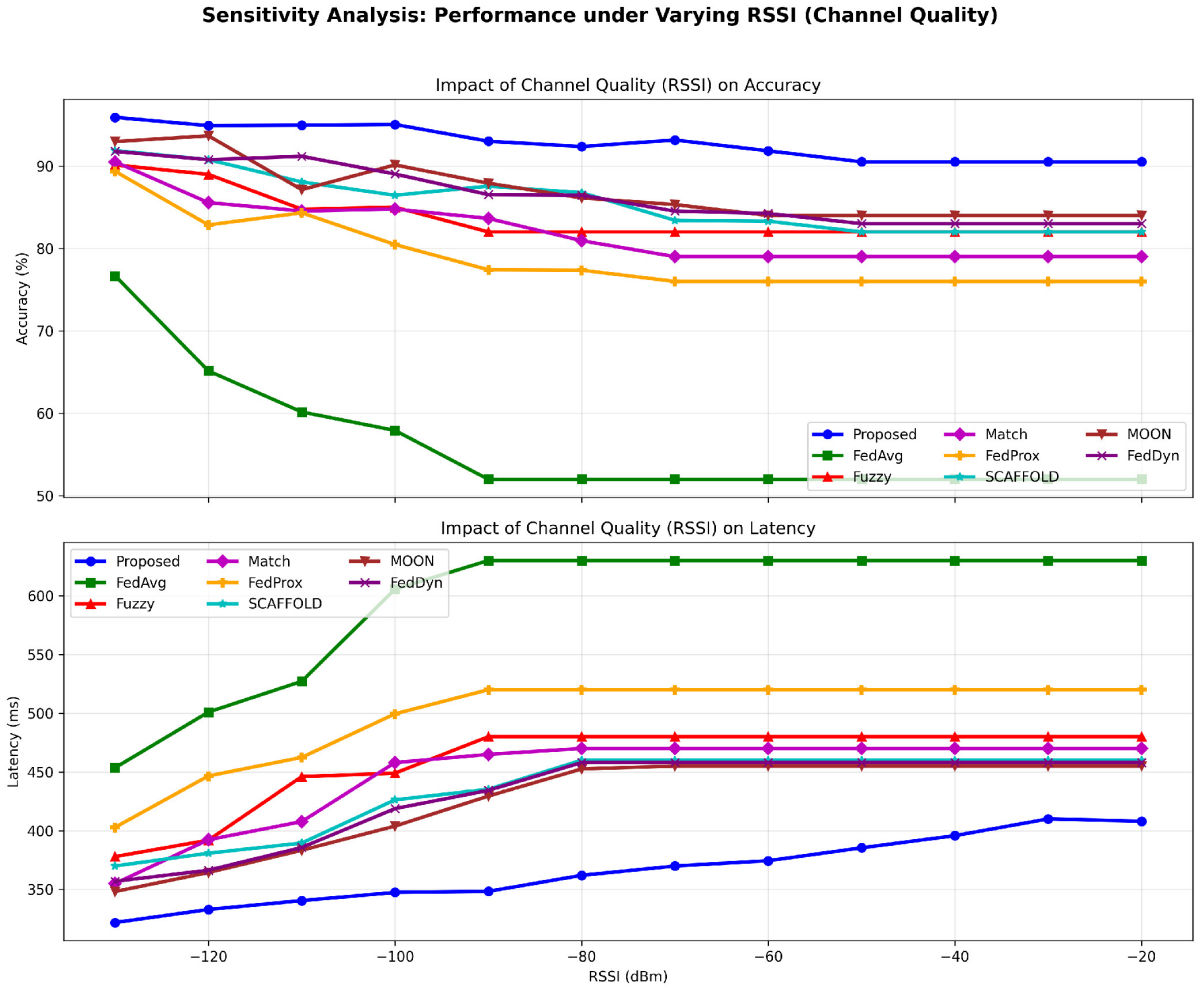
Impact of channel quality (RSSI) on accuracy and latency across all baselines.

While Figures 3–9 demonstrate the superior convergence and final performance of the proposed framework under average channel conditions, Figure 10 further evaluates robustness against varying channel quality. The proposed method maintains accuracy above 90% and limits latency degradation even at low RSSI values thanks to adaptive game-theoretic matching.

The scalability experiments in this paper are limited to a fixed configuration of 50 vehicles and 5 Level-1 aggregators; we have not yet evaluated performance under larger vehicular networks (e.g., 100–200 vehicles), varying RSU density (e.g., 8–12 Level-1 aggregators), or explicit communication failure modeling beyond the RSSI-driven degradation already tested above. Investigating these dimensions—particularly how the communication-overhead trade-off quantified in Section 4.4 shifts as vehicle count grows relative to a fixed number of aggregators—is an important direction for future work and would further substantiate the framework's practical scalability claims.

### 4.6 Discussion

The proposed hierarchical federated learning framework advances privacy-preserving AI for IoV by synergistically integrating FedNova normalization, Reward-Based Payoff Strategy (RBPS) for incentive-aware selection, game-theoretic stable matching, and QKD-assisted secure aggregation. It achieves strong overall performance (94.8% accuracy, 33% faster convergence, and 29% lower latency compared to FedAvg) on the VeReMi misbehavior detection dataset, with DLG leakage reduced to 5.0%.

Compared to recent state-of-the-art baselines, our framework demonstrates clear advantages. It outperforms SCAFFOLD (control variate method) by achieving higher accuracy and faster convergence while imposing a substantially lighter communication load on the central aggregator through hierarchical aggregation (Section 4.4), even though aggregate network-wide bytes are not reduced. Against MOON (model-contrastive learning), our approach shows better privacy protection and resource efficiency thanks to the RBPS incentive mechanism and stable matching. Similarly, it surpasses FedDyn in both accuracy and latency under severe non-IID conditions, primarily due to FedNova's normalization and the mobility-aware matching strategy. These results highlight that the tight integration of components delivers complementary gains that exceed those of individual advanced methods.

From a practical standpoint, the reduction in convergence rounds enables model updates 5–10 min faster per cycle, which is vital for adapting to dynamic traffic and evolving attack scenarios. The latency improvement to 320 ms supports time-sensitive intelligent transportation applications where delays exceeding 400 ms can compromise safety. These gains, combined with superior resource efficiency and privacy protection, demonstrate the framework's suitability for real-world deployment in latency-critical IoV systems.

Although the current evaluation focuses on the VeReMi misbehavior detection dataset (a standard benchmark in the field), future work will include cross-validation on additional vehicular datasets to further demonstrate generalizability.

### 4.7 Limitations

Several limitations persist. Quantum components are simulated classically using Qiskit, without accounting for NISQ noise or real hardware evaluation. The experiments are limited to 50 vehicles, whereas real IoV deployments involve larger scales. Incentive mechanisms are simulated without economic validation, and mobility and dropout modeling are simplified. The fairness properties of RBPS and vehicle-aggregator matching (Section 3.3) are argued structurally rather than measured empirically (e.g., via a quantitative fairness index such as Jain's index computed across vehicles and rounds), and the long-term participation incentive is likewise a design property that has not yet been validated against observed participation or payoff stability over extended training runs; both are natural targets for future quantitative evaluation. These factors constrain the direct transferability of the results to real-world scenarios.

#### 4.7.1 Adversarial robustness: malicious participants and Byzantine attacks

The privacy evaluation in this paper (Section 3, “Privacy protection is evaluated under a semi-honest adversary model”) assumes aggregators follow the protocol correctly while potentially attempting to infer private information; it does not model actively malicious *participants*. In particular, the FedNova-weighted aggregation used at every tier (Equations 4–6) is a weighted-averaging scheme and, like standard FedAvg, provides no inherent robustness against a vehicle submitting a poisoned or adversarially crafted update, nor against colluding malicious aggregators. This is a meaningful limitation given the paper's application domain: a framework for detecting Sybil and misbehavior attacks is not itself hardened against a Sybil or malicious participant attacking the federated learning process. We note that Byzantine-robust aggregation rules (e.g., coordinate-wise median, trimmed mean, or Multi-Krum-style distance-based filtering, the latter used for exactly this purpose by the recently discussed FedIoV framework Heidari et al., 2026c) are a natural extension, and replacing or augmenting the FedNova aggregation step at Level-1 and Level-2 with such a rule is left as future work.

#### 4.7.2 Computational overhead at the vehicle

The local training burden assumed at each vehicle (Table 3: 5 local epochs on a 3-convolutional/2-fully-connected network) has not been validated against the compute constraints of real connected-vehicle onboard or ADAS-class hardware; Section 4.4's communication-overhead analysis and the implementation-cost discussion earlier in this section address network and edge-server cost, but not vehicle-side compute feasibility, which we leave for future validation on representative automotive hardware.

Additionally, the current evaluation assumes no client dropouts per round. Future work will extend the framework with adaptive re-matching mechanisms, partial aggregation strategies, and predictive mobility models using extended SUMO traces to better handle dynamic topology changes inherent in real IoV environments.

#### 4.7.3 QKD-assisted privacy: deployable mechanisms versus future quantum-enabled implementations

It is important to distinguish clearly what this framework's privacy mechanism relies on today from what depends on future quantum hardware maturing.

*Deployable today*: classical gradient masking, which is what actually obfuscates vehicle updates against the semi-honest aggregators in our evaluation, and classical symmetric-key encryption of the communication channel itself, both of which run on existing RSU/BS and vehicle hardware with no quantum components required.

*Dependent on future quantum-enabled implementations*: the QKD key-establishment step and the exploratory phase-obfuscation circuits are currently executed via classical simulation with Qiskit rather than a physical quantum channel; the measured privacy gains reported in this paper (DLG success rate reduced to 5.0%) come from the classical masking layer and would hold even if the key-establishment step used ordinary classical key exchange instead of simulated QKD. QKD's added value in this design is therefore forward-looking — information-theoretic key security once real quantum hardware is available — rather than a source of the privacy gains already measured.

#### 4.7.4 Practical deployment implications for vehicular networks

Beyond the classical-simulation caveat above, there is a more fundamental physical obstacle to deploying real QKD on the vehicle-to-RSU link specifically. QKD requires an uninterrupted optical path between endpoints, realized either over fiber or via a free-space line-of-sight link; fiber is obviously unavailable to a moving vehicle, and free-space QKD to a mobile endpoint remains an active but comparatively early-stage research area, requiring precise real-time tracking and pointing between transmitter and receiver and remaining sensitive to atmospheric turbulence, weather, and line-of-sight obstruction from buildings or other vehicles (Qi et al., 2015). This stands in contrast to QKD over *fixed* terrestrial infrastructure, where trusted-node fiber networks are already at a national-scale planning and deployment stage in some regions (e.g., the EU's EuroQCI initiative) (Raubitzek et al., 2026). This distinction maps naturally onto the two different link types already present in our architecture (Section 3.1): the Level-1-to-Level-2-to-SDNC backhaul is assumed to run over wired infrastructure, which is exactly the setting where terrestrial trusted-node QKD is closest to practical deployment today, whereas the vehicle-to-RSU last hop is wireless and mobile, where real QKD deployment would currently require either a stationary hand-off point (e.g., a vehicle briefly docking or pausing at a fixed roadside QKD terminal) or a mature mobile free-space QKD capability that does not yet exist at vehicular scale. A realistic near-term deployment path is therefore a hybrid one: QKD-secured keys on the fixed RSU-cluster-to-SDNC backhaul where the technology is comparatively mature, combined with classical, standards-based authenticated key exchange (e.g., certificate-based V2X security such as IEEE 1609.2) on the vehicle-to-RSU hop until mobile QKD matures—rather than the uniform QKD-everywhere picture that a reader could otherwise take from the framework's name. The QKD-assisted privacy mechanism is currently evaluated through classical simulation, with privacy gains quantified primarily via the Deep Leakage from Gradients (DLG) attack. While this provides a strong baseline, future work will incorporate evaluation against additional attacks and formal differential privacy guarantees when real quantum hardware becomes available.

#### 4.7.5 Practical deployment considerations

We discuss four aspects relevant to deploying this framework on real software-defined vehicular infrastructure.

##### 4.7.5.1 Integration with existing infrastructure

The Level-1 and Level-2 aggregation, RBPS scoring, and matching logic are all software components that could run as applications on existing Multi-access Edge Computing (MEC) nodes already being deployed at RSUs and base stations under current 5G rollouts, following the standard ETSI MEC architecture. Where MEC-capable compute is already present at a roadside site, deploying this framework requires adding a software module rather than new physical infrastructure; where it is not, the incremental hardware need is modest edge-server compute rather than specialized equipment, since Level-1/Level-2 aggregation and RBPS/matching computation (Section 3.8) are far lighter workloads than the FedNova model aggregation itself, which is a standard weighted-averaging operation well within the capability of typical MEC hardware.

##### 4.7.5.2 Communication standards

The framework operates at the application layer and does not require a new physical- or MAC-layer standard. Vehicle-to-RSU messages (masked model updates, matching proposals) can be carried as application payloads over existing V2X communication stacks—IEEE 1609.x (WAVE/DSRC), ETSI ITS-G5, or 3GPP C-V2X/5G NR-V2X (Uu or PC5 sidelink)—in the same way any other ITS application-layer message is encapsulated today, rather than requiring modification to these standards themselves.

##### 4.7.5.3 Compatibility with 6G

Ongoing 6G vehicular research emphasizes AI-native network design, integrated sensing and communication, and network slicing for differentiated quality-of-service classes; a federated-learning-based framework such as this one is a natural fit for that direction rather than a legacy approach requiring adaptation. Practically, 6G's anticipated higher bandwidth and lower latency would also directly ease the communication-overhead trade-off quantified in Section 4.4: the additional bytes introduced by hierarchical relay hops (+14% vs. a flat topology) would be comparatively cheaper to carry on higher-bandwidth 6G links, while lower air-interface latency would further reduce the end-to-end delay contributed by the vehicle-to-RSU hop specifically.

##### 4.7.5.4 Implementation costs

Costs are concentrated in two places rather than distributed uniformly across the system. Software integration onto existing MEC-enabled RSUs is comparatively low-cost, since it adds a processing module rather than new radio hardware. The more significant cost driver is the QKD-secured backhaul discussed above (Section 3.1): fiber-based trusted-node QKD equipment is costly and would realistically be justified only on the fixed RSU-cluster-to-SDNC backhaul rather than deployed per-vehicle or per-RSU, keeping quantum-related hardware costs bounded to core infrastructure rather than scaling with fleet size. A full cost-benefit analysis against a real telecom operator's existing RSU/MEC deployment plan is beyond the scope of this simulation-based study and is noted as future work below. The proposed hierarchical framework demonstrates strong potential for real-world deployment in latency-critical IoV applications. The three-layer architecture with edge-level preliminary aggregation and matching reduces the central aggregator's direct communication load, even though it does not reduce aggregate network-wide bytes (Section 4.4). However, scalability to large vehicle fleets and the economic feasibility of incentive mechanisms require further large-scale validation using real 5G/6G V2X testbeds.

## 5 Conclusion

This paper presented a hierarchical federated learning framework for the Internet of Vehicles that integrates FedNova normalization, a Reward-Based Payoff Strategy (RBPS) for incentive-aware client selection, game-theoretic stable matching, and QKD-assisted secure aggregation. The core novelty lies in the tight synergistic integration of these components, creating a closed feedback loop between incentives, selection, and pairing within a three-layer software-defined vehicular fog architecture.

Extensive simulations on the VeReMi dataset demonstrate that the proposed approach achieves 94.8% classification accuracy, 33% faster convergence, and 29% lower latency compared to strong baselines, with significantly improved privacy protection. Ablation studies and sensitivity analyses confirm the complementary contributions of each component and the framework's robustness under varying channel conditions.

While the current work makes simplifying assumptions (e.g., no dropouts per round) and relies on classical simulation for quantum components, it provides a solid foundation for privacy-preserving, latency-aware federated learning in dynamic IoV environments. Beyond the specific limitations already discussed in Section 4.6, we highlight seven concrete directions for future work. *Personalized federated learning* could allow individual vehicles to retain locally adapted model components rather than a single shared global model, potentially easing the accuracy-versus-heterogeneity trade-offs observed under severe non-IID conditions (Table 5). *Asynchronous aggregation* would relax the synchronous, per-round design adopted throughout this paper (Section 3.1), allowing slower vehicles or aggregators to contribute without blocking the round, at the cost of additional staleness-handling complexity. *Digital-twin-assisted vehicular intelligence* could extend the SUMO/Veins mobility modeling already used here into a continuously updated virtual representation of the road network, potentially improving the accuracy of the mobility-triggered re-matching described in Section 3.1. *Reinforcement-learning-based client scheduling* is a natural alternative or complement to the RBPS payoff and matching mechanism proposed here, learning selection policies directly from observed reward signals rather than a fixed weighted formula. *Post-quantum cryptography* offers a complementary near-term path to the QKD-based privacy mechanism discussed in Section 4.6: unlike QKD, post-quantum cryptographic schemes run on existing classical hardware and would not face the vehicle-mobility deployment obstacle identified there, making them a plausible interim option while mobile QKD matures. *Foundation models for vehicular edge intelligence* could replace the compact convolutional architecture used in this paper (Table 3) with a pre-trained, more broadly capable backbone, provided its computational demands remain compatible with the vehicle-side resource constraints noted as a limitation above. Finally, *real-world field deployment*—on real 5G/6G V2X testbeds, with real client dropouts, real economic incentive validation, and the practical integration path discussed in Section 4.6—remains the ultimate test of the framework's claims beyond simulation.

The proposed framework represents a meaningful step toward trustworthy and efficient artificial intelligence systems for next-generation intelligent transportation.

## Data Availability

The original contributions presented in the study are included in the article/supplementary material, further inquiries can be directed to the corresponding author.
